# Supplementary material for: Probing the genomic and proteomic basis of encystment in Oxytricha granulifera
Source: mSystems. 2026 Apr 27;11(5):e01757-25. doi: 10.1128/msystems.01757-25 (PMC13185551; doi:10.1128/msystems.01757-25)
Supplement: Supplemental Material — Fig. S1 to S3; Tables S1 to S3. [file msystems.01757-25-s0001.docx]

**Supplementary Materials**

**
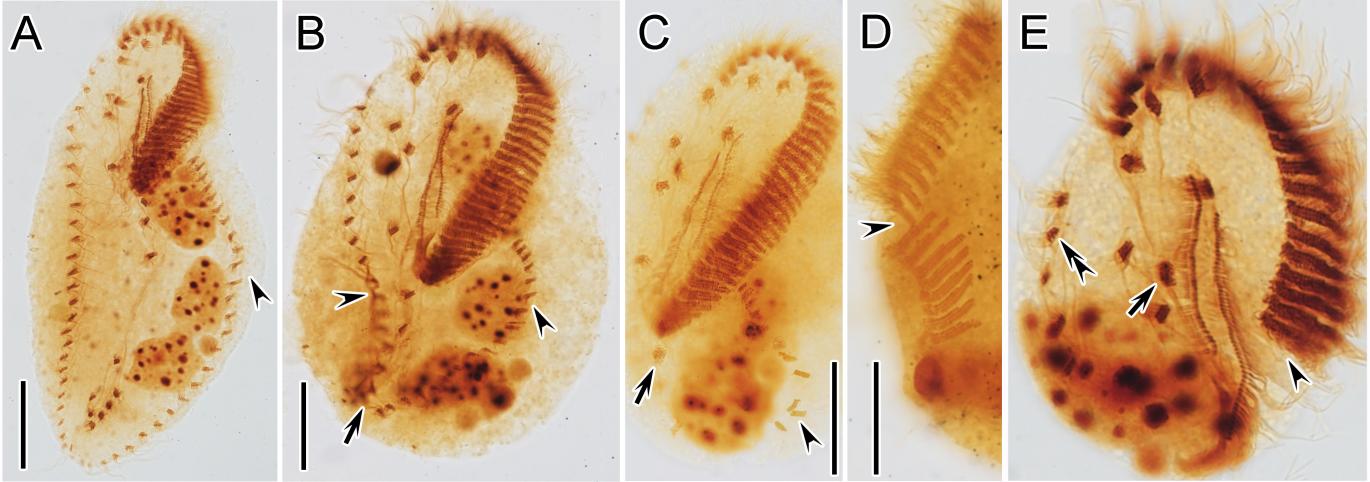
**

**Figure S1. Protargol staining showing the ciliature dedifferentiation during the early stages of encystment of *Oxytricha granulifera*.** (A) Body and left marginal row (arrowhead) begin to twist. (B) The decrease of transverse cirri and pre-transverse ventral cirri (arrow), and the distinctively twisted marginal rows (arrowheads). (C) The dedifferentiated left marginal cirri and only one postoral ventral cirrus remains (arrow). (D, E) The posterior portion of adoral membranelles break down (arrowhead) and only few right marginal cirri (double-arrowhead) and three frontal-ventral cirri (arrow) remains. Scale bars: 20 μm.

**
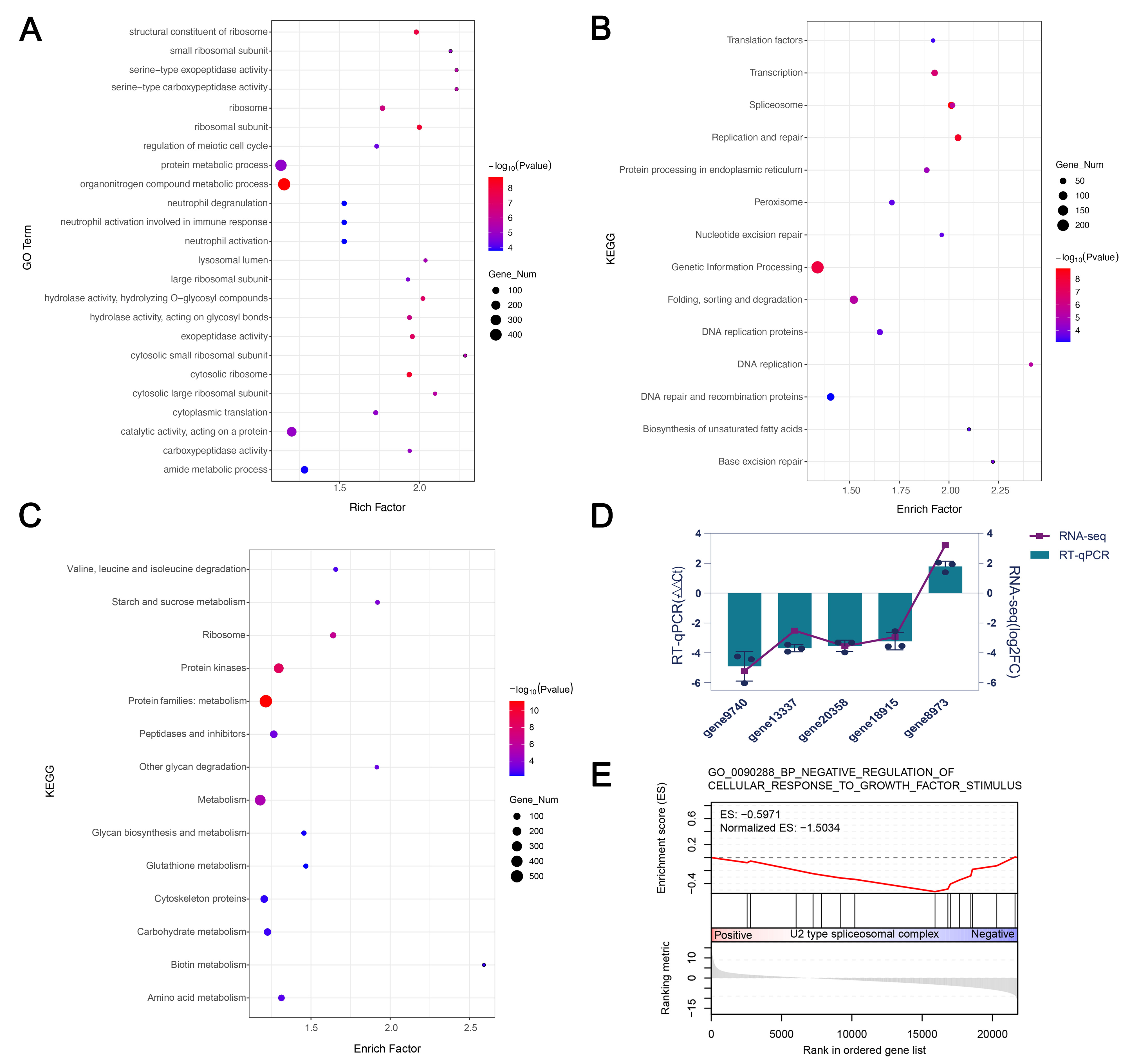
**

**Figure S2. GO and KEGG annotation of differentially expressed genes in transcriptome.** (A) GO enrichment of downregulated genes. (B) KEGG enrichment of upregulated genes. (C) KEGG enrichment of downregulated genes. (D) Expression pattern of genes as determined by RNA-seq and RT-qPCR. (E) GSEA analysis of regulation of cellular response to growth factor stimulus.


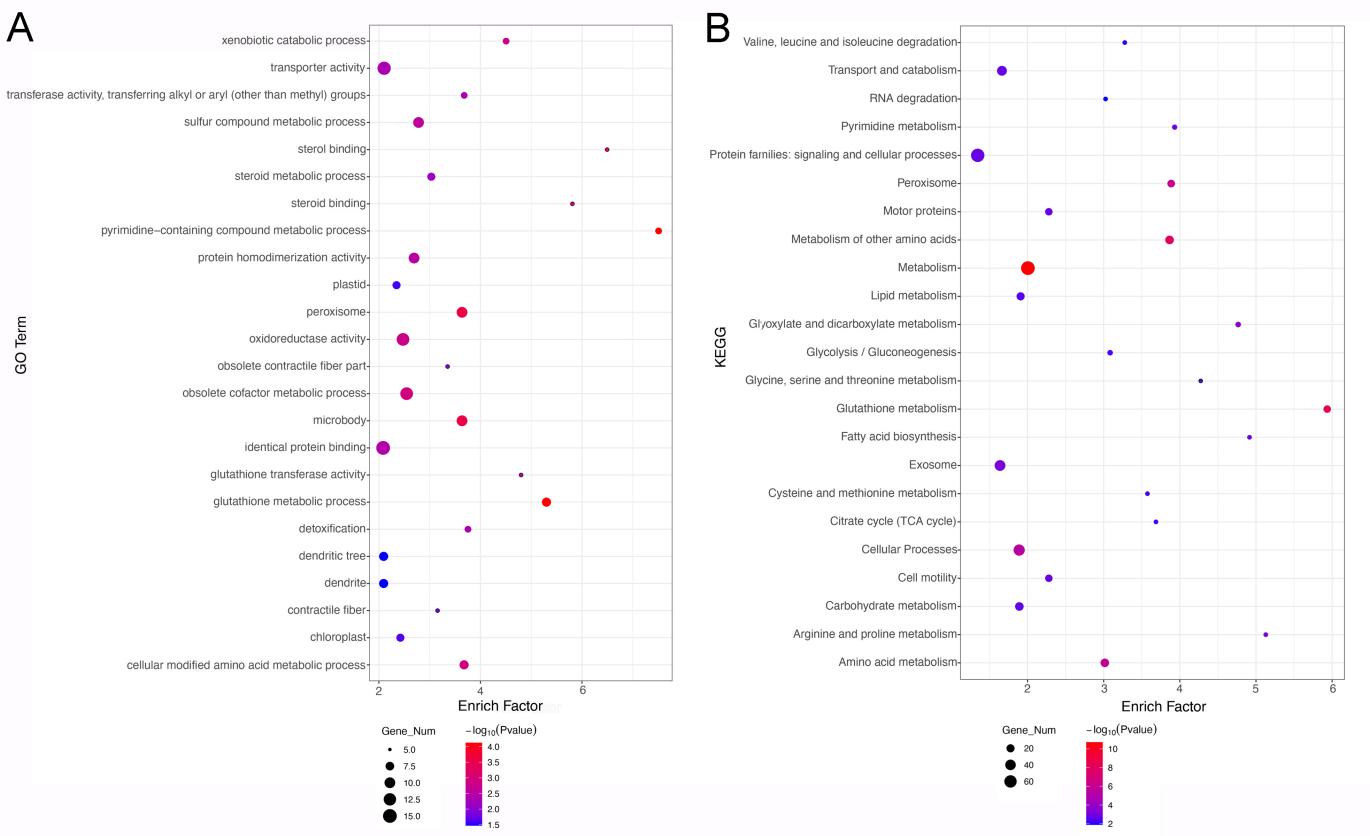


**Figure S3. GO and KEGG annotation of differentially expressed genes in proteome.** (A) GO enrichment of downregulated genes. (B) KEGG enrichment of downregulated genes.

**Table S1. Information of genome assemblies analyzed in the current study.**

| **Class** | **Subclass** | **Order** | Species | Data source |
| --- | --- | --- | --- | --- |
| Oligohymenophorea | Scuticociliatia | Philasterida | *Uronema marinum* | Wang, et al. (2018) |
| Oligohymenophorea | Scuticociliatia | Philasterida | *Pseudocohnilembus persalinus* | Xiong, et al. (2015) |
| Oligohymenophorea | Hymenostomatia | Tetrahymenida | *Tetrahymena thermophila* | TGD (https://tet.ciliate.org/) |
| Oligohymenophorea | Hymenostomatia | Tetrahymenida | *Tetrahymena borealis* | TGD (https://tet.ciliate.org/) |
| Oligohymenophorea | Peniculia | Peniculida | *Paramecium teraurelia* | ParameciumDB (https://paramecium.i2bc.paris-saclay.fr/) |
| Oligohymenophorea | Peniculia | Peniculida | *Paramecium biaurelia* | ParameciumDB (https://paramecium.i2bc.paris-saclay.fr/) |
| Colpodea | - | Cyrtolophosidida | *Platyophrya macrostoma* | Gentekaki, et al. (2017) |
| Colpodea | - | Cyrtolophosidida | *Aristerostoma* sp. | MMETSP0125 (MMETSP) |
| Spirotrichea | Choreotrichia | Tintinnida | *Favella taraikaensis* | MMETSP0434 (MMETSP) |
| Spirotrichea | Choreotrichia | Tintinnida | *Schmidingerella arcuata [Favella ehrenbergii]* | Gentekaki, et al. (2017) |
| Spirotrichea | Choreotrichia | Choreotrichida | *Strombidinopsis acuminatum* | Gentekaki, et al. (2017) |
| Spirotrichea | Oligotrichia | Strombidiida | *Strombidium rassoulzadegani* | MMETSP0449 (MMETSP) |
| Spirotrichea | Oligotrichia | Strombidiida | *Strombidium sculcatum* | Chen, et al. (2016) |
| Spirotrichea | Oligotrichia | Strombidiida | *Strombidium inclinatum* | Gentekaki, et al. (2017) |
| Spirotrichea | Stichotrichia | Sporadotrichida | *Oxytricha trifallax* | OxyDB (https://oxy.ciliate.org/) |
| Spirotrichea | Stichotrichia | Sporadotrichida | *Oxytricha granulifera* | Current work |
| Spirotrichea | Stichotrichia | Sporadotrichida | *Stylonychia lemnae* | Aeschlimann, et al. (2014) |
| Spirotrichea | Stichotrichia | Urostylida | *Pseudokeronopsis* sp. 1 | MMETSP1396 (MMETSP) |
| Spirotrichea | Stichotrichia | Urostylida | *Pseudokeronopsis flava* | Zheng, et al. (2022) |
| Spirotrichea | Stichotrichia | Urostylida | *Pseudokeronopsis carnea* | Zheng, et al. (2022) |
| Spirotrichea | Stichotrichia | Urostylida | *Pseudokeronopsis* sp. 2 | MMETSP0211 (MMETSP) |
| Spirotrichea | Hypotrichia | Euplotida | *Euplotes harpa* | Gentekaki, et al. (2017) |
| Spirotrichea | Hypotrichia | Euplotida | *Euplotes octocarinatus* | EOGD (http://ciliates.ihb.ac.cn/database/home/#eo) |
| Spirotrichea | Hypotrichia | Euplotida | *Euplotes vannus* | EvanDB (https://evan.ciliate.org/) |
| Spirotrichea | Hypotrichia | Euplotida | *Euplotes crassus* | Vinogradov, et al. (2012) |
| Litostomatea | Trichostomatia | Entodiniomorphida | *Entodinium caudatum* | Park T et al. (2021) |
| Litostomatea | Haptoria | Pleurostomatida | *Litonotus pictus* | Gentekaki, et al. (2017) |
| Protocruziea | Protocruziidia | Protocruziida | *Protocruzia adherens* | Gentekaki, et al. (2017) |
| Heterotrichea | - | Heterotrichida | *Climacostomum virens* | MMETSP1397 (MMETSP) |
| Heterotrichea | - | Heterotrichida | *Blepharisma japonicum* | MMETSP1395 (MMETSP) |
| Heterotrichea | - | Heterotrichida | *Fabrea salina* | MMETSP1345 (MMETSP) |
| Heterotrichea | - | Heterotrichida | *Sentor coeruleus* | Slabodnick, et al. (2017) |

**Table S2. List of MT-A70 proteins.**

| **Species** | **NCBI Accession No.** | **Protein ID** |
| --- | --- | --- |
| *Absidia repens* | ORZ21199.1 | hypothetical protein BCR42DRAFT_368925 |
| *Absidia repens* | ORZ22096.1 | hypothetical protein BCR42DRAFT_458918 |
| *Absidia repens* | ORZ15132.1 | MT-A70-like protein, partial |
| *Absidia repens* | ORZ06213.1 | MT-A70-domain-containing protein |
| *Acanthamoeba castellanii* | ELR12528.1 | MT-A70 protein |
| *Acanthamoeba castellanii* | ELR14023.1 | MTA70 family protein |
| *Acanthamoeba castellanii* | ELR15231.1 | MT-A70 protein |
| *Acanthamoeba castellanii* | ELR23875.1 | Putative N6adenosine-methyltransferase |
| *Afipia* | WP_009339935.1 | MULTISPECIES: S-adenosylmethionine-binding protein |
| *Anaeromyces robustus* | ORX85672.1 | hypothetical protein BCR32DRAFT_216900 |
| *Anaeromyces robustus* | ORX86973.1 | MT-A70-domain-containing protein |
| *Arabidopsis thaliana* | CAB39622.1 | putative protein |
| *Arabidopsis thaliana* | NP_564080.1 | Methyltransferase MT-A70 family protein |
| *Arabidopsis thaliana* | O82486.2 | N6-adenosine-methyltransferase MT-A70-like |
| *Basidiobolus meristosporus* | ORX98979.1 | allantoinase |
| *Basidiobolus meristosporus* | ORX92345.1 | MT-A70-domain-containing protein |
| *Blastocystis hominis* | CBK22092.2 | unnamed protein product |
| *Blastocystis hominis* | CBK23014.2 | unnamed protein product |
| *Caenorhabditis elegans* | Q09956.1 | DNA N6-methyl methyltransferase |
| *Candidatus Entotheonella factor* | ETW92643.1 | S-adenosylmethionine-binding protein |
| *Chlamydomonas reinhardtii* | XP_001691478.1 | predicted protein |
| *Chlamydomonas reinhardtii* | XP_001698523.1 | predicted protein, partial |
| *Chlamydomonas reinhardtii* | PNW77799.1 | hypothetical protein CHLRE_10g452300v5 |
| *Chlamydomonas reinhardtii* | PNW82819.1 | hypothetical protein CHLRE_06g295600v5 |
| *Chlamydomonas reinhardtii* | PNW88915.1 | hypothetical protein CHLRE_01g050600v5 |
| *Chlamydomonas reinhardtii* | XP_042924083.1 | CHLREDRAFT_174824 |
| *Clostridioides difficile* | WP_009320301.1 | DNA methyltransferase |
| *Danio rerio* | AAH67182.1 | Methyltransferase like 3 |
| *Danio rerio* | Q6NZ22.1 | Methyltransferase-like protein 14 |
| *Danio rerio* | XP_689178.3 | methyltransferase-like protein 4 isoform X1 |
| *Danio rerio* | F1R777.1 | N6-adenosine-methyltransferase subunit METTL3 |
| *Devosia riboflavina* | KFL31466.1 | DNA methyltransferase |
| *Drosophila melanogaster* | NP_650573.1 | methyltransferase like 4 |
| *Drosophila melanogaster* | Q9VCE6.1 | N6-adenosine-methyltransferase MT-A70-like protein |
| *Drosophila melanogaster* | Q9VLP7.1 | Methyltransferase-like protein 14 |
| *Escherichia coli* | ESK34829.1 | hypothetical protein G966_02949 |
| *Escherichia coli* | AIF94871.1 | Adenine DNA methyltransferase, phage-associated |
| *Ethanoligenens harbinense* | WP_013485562.1 | S-adenosylmethionine-binding protein |
| *Granulibacter bethesdensis* | AHJ63281.1 | Adenine-specific methyltransferase |
| *Hesseltinella vesiculosa* | ORX58127.1 | MT-A70-domain-containing protein |
| *Hesseltinella vesiculosa* | ORX43344.1 | MT-A70-domain-containing protein |
| *Homo sapiens* | NP_066012.1 | N6-adenosine-methyltransferase non-catalytic subunit |
| *Homo sapiens* | AAI11021.1 | Methyltransferase like 4 |
| *Homo sapiens* | NP_073751.3 | methyltransferase-like protein 4 isoform 1 |
| *Homo sapiens* | NP_062826.2 | N6-adenosine-methyltransferase catalytic subunit |
| *Klebsiella aerogenes* | WP_032715146.1 | adenine methylase |
| *Klebsiella pneumoniae* | AIA43360.1 | DNA methyltransferase |
| *Linderina pennispora* | ORX69627.1 | MT-A70-domain-containing protein |
| *Lobosporangium transversale* | XP_021879935.1 | MT-A70-domain-containing protein |
| *Lobosporangium transversale* | XP_021880122.1 | MT-A70-domain-containing protein |
| *Methylococcus capsulatus* | WP_017364718.1 | S-adenosylmethionine-binding protein |
| *Mus musculus* | XP_011245012.1 | PREDICTED: methyltransferase-like protein 4 isoform X2 |
| *Mus musculus* | NP_964000.2 | N6-adenosine-methyltransferase non-catalytic subunit |
| *Mus musculus* | Q8C3P7.2 | N6-adenosine-methyltransferase subunit METTL3 |
| *Mycobacteroides abscessus* | WP_016343787.1 | adenine-specific DNA methyltransferase |
| *Nitratireductor basaltis* | KFB10357.1 | Adenine-specific methyltransferase |
| *Oxytricha granulifera* | N/A | gene9414 |
| *Oxytricha granulifera* | N/A | gene13924 |
| *Oxytricha granulifera* | N/A | gene15252 |
| *Oxytricha granulifera* | N/A | gene4722 |
| *Oxytricha trifallax* | EJY88228.1 | MT-A70 family protein |
| *Oxytricha trifallax* | EJY79437.1 | MT-A70 family protein |
| *Paramecium tetraurelia* | CAI39082.1 | Putative N6-adenosine-methyltransferase with ZZ-type zinc-binding domains |
| *Paramecium tetraurelia* | CAI39084.1 | Putative N6-adenosine-methyltransferase with ZZ-type zinc-binding domains |
| *Paramecium tetraurelia* | CAI39085.1 | Putative N6-adenosine-methyltransferase with ZZ-type zinc-binding domains |
| *Paramecium tetraurelia* | CAI39087.1 | Putative mRNA N6-adenosine-methyltransferase |
| *Paramecium tetraurelia* | XP_001462525.1 | uncharacterized protein GSPATT00027481001 |
| *Paramecium tetraurelia* | XP_001424228.1 | uncharacterized protein GSPATT00027862001 |
| *Paramecium tetraurelia* | XP_001429706.1 | uncharacterized protein GSPATT00005554001 |
| *Paramecium tetraurelia* | XP_001432030.1 | uncharacterized protein GSPATT00034109001 |
| *Paramecium tetraurelia* | XP_001435819.1 | uncharacterized protein GSPATT00037207001 |
| *Paramecium tetraurelia* | XP_001453129.1 | uncharacterized protein GSPATT00019450001 |
| *Paramecium tetraurelia* | XP_001457964.1 | uncharacterized protein GSPATT00003428001 |
| *Paramecium tetraurelia* | XP_001458901.1 | uncharacterized protein GSPATT00024232001 |
| *Paramecium tetraurelia* | EAS04360.2 | hypothetical protein TTHERM_00301770 |
| *Paramecium tetraurelia* | EAR84411.3 | MT-a70 family protein |
| *Paramecium tetraurelia* | EAS00013.2 | N6-adenosine-methyltransferase 70 kDa subunit |
| *Paramecium tetraurelia* | EAR99483.3 | MT-a70 family protein |
| *Paramecium tetraurelia* | EAR99108.2 | MT-A70 family protein |
| *Paramecium tetraurelia* | EAR90090.2 | methyltransferase MT, putative |
| *Physcomitrium patens* | PNR63074.1 | hypothetical protein PHYPA_001499 |
| *Physcomitrium patens* | XP_024378153.1 | methyltransferase-like protein 1 |
| *Physcomitrium patens* | XP_024386705.1 | methyltransferase-like protein 2 isoform X1 |
| *Piromyces finnis* | ORX52920.1 | MT-A70 protein |
| *Plasmodium falciparum* | KNC35464.1 | MT-A70 family protein |
| *Plasmodium falciparum* | KNG77137.1 | mRNA (N6-adenosine)-methyltransferase |
| *Pseudocohnilembus persalinus* | N/A | PpAMT1 |
| *Pseudocohnilembus persalinus* | N/A | PpAMT7 |
| *Reticulomyxa filosa* | ETO06258.1 | MT-A70 family protein, partial |
| *Reticulomyxa filosa* | ETO32567.1 | hypothetical protein RFI_04550, partial |
| *Rhizobium phaseoli* | WP_016734132.1 | DNA methyltransferase |
| *Rhizobium undicola* | WP_027488351.1 | S-adenosylmethionine-binding protein |
| *Rothia* | WP_023133224.1 | MULTISPECIES: MT-A70 protein |
| *Saccharomyces cerevisiae* | CAA96904.1 | IME4 |
| *Saccharomyces cerevisiae* | KZV12799.1 | KAR4 |
| *Saccharomyces cerevisiae* | NP_009876.1 | Kar4p |
| *Saccharomyces cerevisiae* | AJR96662.1 | Ime4p |
| *Sus scrofa* | XP_020951799.1 | methyltransferase-like protein 4 isoform X1 |
| *Sus scrofa* | XP_003129279.3 | N6-adenosine-methyltransferase subunit METTL14 |
| *Sus scrofa* | XP_003128628.1 | N6-adenosine-methyltransferase 70 kDa subunit |
| *Syncephalastrum racemosum* | ORY94237.1 | MT-A70-domain-containing protein |
| *Syncephalastrum racemosum* | ORZ00623.1 | MT-A70-domain-containing protein |
| *Tetrahymena thermophila* | EAS02129.1 | MT-a70 family protein |
| *Xanthobacter autotrophicus* | WP_012115592.1 | MT-A70 family protein |
| *Xenopus laevis* | XP_018079135.1 | PREDICTED: methyltransferase-like protein 4 |
| *Xenopus laevis* | XP_018099083.1 | N6-adenosine-methyltransferase subunit METTL14 isoform X2 |
| *Xenopus laevis* | NP_001084701.1 | methyltransferase like 3 L homeolog |
| *Xylella fastidiosa* | WP_027700599.1 | S-adenosylmethionine-binding protein |

**Table S3. Primer sequences and gene information for RT-qPCR validation.**

|  | **gene_ID** | **Prime_R** | **Prime_F** | **q-PCR(-ΔΔCt)** | | | **RNA-seq (log2FC)** | **RNA-seq (p-value)** |
| --- | --- | --- | --- | --- | --- | --- | --- | --- |
| Gene1 | gene9740 | GCCAAAGTATGGGACCTCCT | GGAGTTCGGTGTATGCTGTGT | -4.2416 | -4.4266 | -6.0336 | -5.5072 | 2.1412E-06 |
| Gene2 | gene13337 | GACATATTCGGAAGCCCGGT | TCTTGCCACAGATATGCCACT | -3.7056 | -3.9216 | -3.4516 | -2.7925 | 2.2179E-03 |
| Gene3 | gene20358 | TTAAGCAGCTGGCTGCACTT | AAGCTTAAAGGGAGCGGTTCT | -3.3110 | -3.9640 | -3.3210 | -3.8221 | 9.4969E-03 |
| Gene4 | gene18915 | TGGAGAAAAGCATGGACAAGGA | CCAACGAACTTTGTGCCGTT | -3.5450 | -3.5700 | -2.5560 | -3.1997 | 3.4476E-05 |
| Gene5 | gene8973 | GGTACAGTTGCTGTGCCTGG | GTGCATCTCACCTCTCAAGCA | 1.9274 | 1.3854 | 2.0344 | 2.9146 | 7.1382E-03 |
